# Comparative Studies of the (Anti) Mutagenicity of *Baccharis* *dracunculifolia* and Artepillin C by the Bacterial Reverse Mutation Test

**DOI:** 10.3390/molecules17032335

**Published:** 2012-02-24

**Authors:** Flávia Aparecida Resende, Carla Carolina Munari, Moacir de Azevedo Bentes Monteiro Neto, Denise Crispim Tavares, Jairo Kenupp Bastos, Ademar Alves da Silva Filho, Eliana Aparecida Varanda

**Affiliations:** 1 Departamento de Ciências Biológicas, Faculdade de Ciências Farmacêuticas de Araraquara, Universidade Estadual Paulista Julio de Mesquita Filho, 14801-902, Araraquara, São Paulo, Brazil; 2 Universidade de Franca, 14404-600, Franca, São Paulo, Brazil; 3 Faculdade de Ciências Farmacêuticas de Ribeirão Preto, Universidade de São Paulo, 14040-903, Ribeirão Preto, São Paulo, Brazil; 4 Departamento de Ciências Farmacêuticas, Faculdade de Farmácia, Universidade Federal de Juiz de Fora, 36035-900, Juiz de Fora, Minas Gerais, Brazil

**Keywords:** *Baccharis dracunculifolia*, artepillin C, Ames test, antimutagenicity, mutagenicity

## Abstract

*Baccharis dracunculifolia* is a plant native from Brazil, commonly known as ‘Alecrim-do-campo’ and ‘Vassoura’ and used in alternative medicine for the treatment of inflammation, hepatic disorders and stomach ulcers. Previous studies reported that artepillin C (ArtC, 3-{4-hydroxy-3,5-di(3-methyl-2-butenyl)phenyl}-2(*E*)-propenoic acid), is the main compound of interest in the leaves. This study was undertaken to assess the mutagenic effect of the ethyl acetate extract of *B. dracunculifolia *leaves (Bd-EAE: 11.4–182.8 µg/plate) and ArtC (0.69–10.99 µg/plate) by the Ames test using *Salmonella typhimurium* strains TA98, TA97a, TA100 and TA102, and to compare the protective effects of Bd-EAE and ArtC against the mutagenicity of a variety of direct and indirect acting mutagens such as 4-nitro-*O*-phenylenediamine, sodium azide, mitomycin C, benzo[*a*]pyrene, aflatoxin B1, 2-aminoanthracene and 2-aminofluorene.The mutagenicity test showed that Bd-EAE and ArtC did not induce an increase in the number of revertant colonies indicating absence of mutagenic activity. ArtC showed a similar antimutagenic effect to that of Bd-EAE in some strains of *S. typhimurium*, demonstrating that the antimutagenic activity of Bd-EAE can be partially attributed to ArtC. The present results showed that the protective effect of whole plant extracts is due to the combined and synergistic effects of a complex mixture of phytochemicals, the total activity of which may result in health benefits.

## 1. Introduction

Plants have been employed in medicine for more than 60,000 years. However, they are frequently employed without scientific knowledge of their biological and therapeutic properties. Recently, scientific study of their chemical properties, biological activities or genotoxic properties has been emerging as a health priority [1].

*Baccharis dracunculifolia *De Candole (Asteraceae) is a native plant from Brazil, commonly known as ‘Alecrim-do-campo’ and ‘Vassoura’. *B. dracunculifolia *is the most important botanical source of green propolis in Southeast Brazil, which is named for its colour [2]. Teas, decoctions and tinctures prepared from the flowering plant are widely used in alternative medicine for the treatment of inflammation, hepatic disorders and stomach ulcers [3]. Thus, in recent years, interest in the chemical composition of *B*. *dracunculifolia*, as well as in its biological activities, has grown substantially [4].

**Figure 1:**
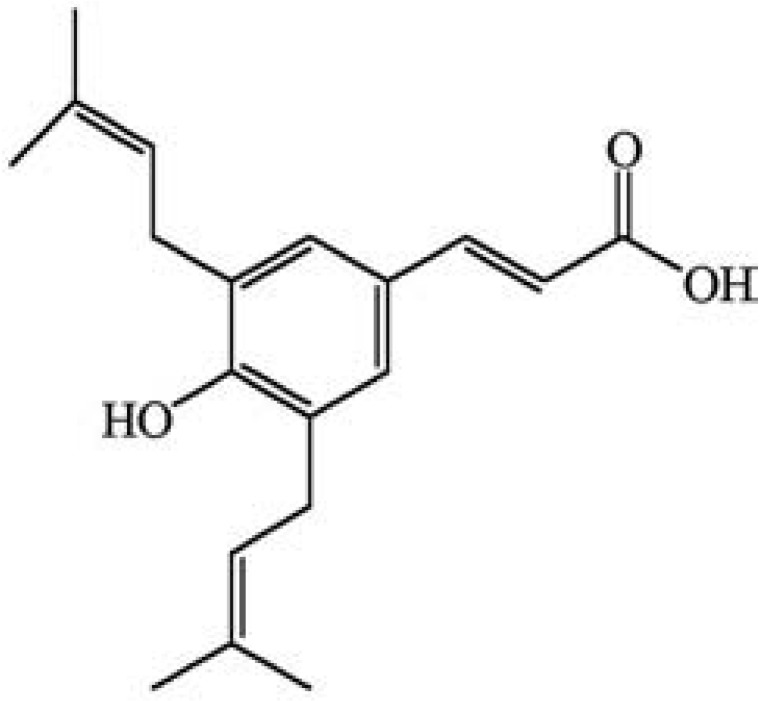
Chemical structure of artepillin C.

**Figure 2:**
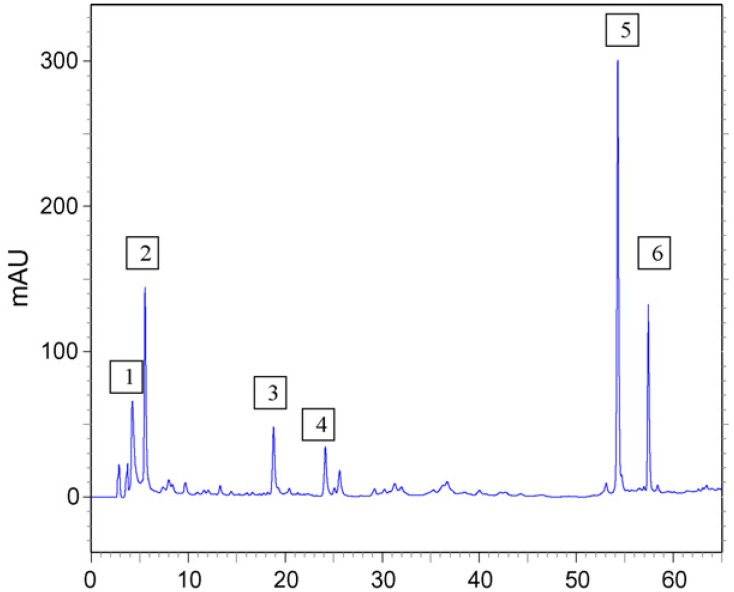
HPLC profile of Bd-EAE. **1**: caffeic acid; **2**: *p*-coumaric acid; **3**: aromadendrin-4′-*O*-methyl ether; **4**: 3-prenyl-*p*-coumaric acid (drupanin); **5**:3,5-diprenyl *p*-coumaric acid (artepillin C); **6**: baccharin.

*B*. *dracunculifolia *exhibits anticariogenic [5], anti-ulcer [2], trypanocidal [6], antimicrobial [3], antimutagenic [7] and immunomodulatory [8] activities. Previous phytochemical studies of this plant have shown that artepillin C (ArtC, 3-{4-hydroxy-3,5-di(3-methyl-2-butenyl)phenyl}-2(*E*)-propenoic acid, Figure 1), a low-molecular weight phenolic compound, is the main compound of interest in the leaves of *B. dracunculifolia *[9], and the HPLC phenolic profile (Figure 2) of *B. dracunculifolia *ethyl acetate extract (Bd-EAE) displays ArtC as the major peak [7]. In addition, Shimizu *et al.* [10] found that ArtC was a highly bioavailable component of Brazilian propolis.

ArtC is considered a candidate for one of the active compounds preventing cancer in propolis [11]. Thus, it is an excellent scavenger of free radicals, similar to catechins [12]. Moreover, ArtC possesses antimicrobial [13], antitumor [11,14], anti-inflammatory [15], apoptosis-inducing [16], immuno-modulatory [8] and antioxidant properties [12,17].

Since some authors claim that most of the biological activities of *B. dracunculifolia* are due to its high levels of prenylated *p*-coumaric acids, mainly ArtC and baccharin [18,19,20], comparative studies of mutagenic and antimutagenic effects would be of great value to determine if *B. dracunculifolia *and ArtC exhibit comparable biological activities. Besides this, the evaluation of the mutagenic effect is an important approach for safe use of the medicinal plants.

Therefore, in this study the Ames test was used to assess the mutagenic effects of the ethyl acetate extract of *B. dracunculifolia *leaves (Bd-EAE) and ArtC and to compare the protective effects of Bd-EAE and ArtC against the mutagenicity of several direct and indirect-acting mutagens such as 4-nitro-*O*-phenylenediamine (NOPD), sodium azide (SA), mitomycin C (MMC), benzo[*a*]pyrene (B[*a*]P), aflatoxin B1 (AFB_1_), 2-aminoanthracene (AA) and 2-aminofluorene (AF).

## 2. Results

### 2.1. Mutagenic Activity

None of the strains of *S. typhimurium*, exposed to different concentrations of Bd-EAE and ArtC, showed two-fold or greater increase in the mean number of revertants as compared to the negative control group, as given in Table 1, which lists the mean number of revertants/plate (M), the standard deviation (SD) and the mutagenic index (MI) after the treatments with Bd-EAE and ArtC, observed in *S. typhimurium *strain TA98, TA100, TA102 and TA97a in the presence (+S9) and absence (-S9) of metabolic activation. The mutagenicity assays show that neither Bd-EAE nor ArtC induced any increase in the number of revertant colonies, indicating the absence of any mutagenic activity.

**Table 1:** Revertants/ plate, standard deviation and mutagenicity index (in brackets) in the strains TA98, TA100, TA102 and TA97a of *S. typhimurium* after treatment with various doses of Bd-EAE and ArtC, with (+S9) and without (−S9) metabolic activation

	Treatments	Number of revertants (M ± SD)/ plate and MI							
		TA 98		TA 100		TA 102		TA 97	
	µg/plate	− S9	+ S9	− S9	+ S9	− S9	+ S9	− S9	+ S9
**Bd-EAE**	**0.00 ^a^**	20 ± 2	27 ± 6	154 ± 10	210 ± 14	255 ± 11	285 ± 24	132 ± 7	263 ± 7
	**11.4**	19 ± 1 (0.9)	26 ± 2 (1.1)	137 ± 8 (0.8)	211 ± 3 (0.9)	282 ± 17 (1.0)	327 ± 10 (1.1)	137 ± 11 (0.9)	280 ± 29 (1.1)
	**22.8**	21 ± 5 (1.0)	32 ± 2 (1.4)	150 ± 11 (0.9)	210 ± 3 (0.9)	272 ± 11 (0.9)	306 ± 6 (1.0)	132 ± 12 (0.9)	292 ± 28 (1.1)
	**45.6**	19 ± 3 (0.9)	32 ± 5 (1.4)	150 ± 8 (0.9)	215 ± 8 (1.0)	283 ± 11 (1.0)	304 ± 9 (1.0)	133 ± 15 (0.9)	275 ± 21 (1.0)
	**91.4**	22 ± 3 (1.0)	30 ± 4 (1.3)	142 ± 11 (0.9)	203 ± 8 (0.9)	234 ± 3 (0.8)	322 ± 13 (1.1)	135 ± 6 (0.9)	255 ± 15 (1.0)
	**182.8**	21 ± 5 (1.0)	23 ± 3 (1.0)	138 ± 13 (0.8)	200 ± 9 (0.9)	196 ± 6 (0.7)	341 ± 13 (1.1)	91 ± 17 (0.6)	238 ± 27 (0.9)
	**Ctrol +**	1347 ± 88 ^b^	1567 ± 115 ^e^	1582 ± 98 ^c^	1456 ± 78 ^e^	1656 ± 60 ^d^	1932 ± 97 ^f^	1766.0 ± 49 ^b^	1789 ± 89 ^e^
**ArtC**	**0.00 ^a^**	20 ± 3	30 ± 3	152 ± 2	164 ± 4	216 ± 2	216 ± 2	150 ± 15	155 ± 2
	**0.69**	21 ± 3 (1.0)	28 ± 3 (0.9)	179 ± 4 (1.2)	171 ± 1 (1.0)	204 ± 4 (0.9)	204 ± 4 (0.9)	207 ± 3 (1.4)	194 ± 3 (1.2)
	**1.37**	24 ± 4 (1.2)	32 ± 1 (1.1)	174 ± 4 (1.1)	171 ± 2 (1.0)	213 ± 3 (1.0)	213 ± 3 (1.0)	221 ± 6 (1.5)	174 ± 1 (1.1)
	**2.75**	28 ± 5 (1.4)	29 ± 3 (1.0)	169 ± 6 (1.1)	144 ± 2 (0.9)	187 ± 5 (0.9)	187 ± 5 (0.9)	246 ± 5 (1.6)	175 ± 4 (1.1)
	**5.49**	29 ± 1 (1.5)	32 ± 2 (1.1)	158 ± 7 (1.0)	153 ± 2 (0.9)	215 ± 5 (1.0)	215 ± 5 (1.0)	207 ± 5 (1.4)	175 ± 1 (1.1)
	**10.99**	27 ± 2 (1.3)	31 ± 1 (1.0)	153 ± 3 (1.0)	129 ± 1 (0.8)	203 ± 6 (0.9)	203 ± 6 (0.9)	192 ± 6 (1.3)	176 ± 3 (1.1)
	**Ctrol +**	842 ± 34 ^b^	1498 ± 33^ e^	2189 ± 73^ c^	1072 ± 25^ e^	1139 ± 42^ d^	1099 ± 27^ f^	1106 ± 23 ^b^	1861 ± 23 ^e^

Bd-EAE = ethyl acetate extract of *B. dracunculifolia *leaves; ArtC = artepillin C; M ± SD = mean and standard deviation; MI = mutagenicity index; ^a^ Negative control: dimethylsulfoxide (DMSO - 50 μL/ plate); Ctrol + = Positive control; ^b^ 4-nitro-*o*-phenylenediamine (NOPD – 10.0 μg/ plate – TA98, TA97a); ^c^ sodium azide (1.25 μg/ plate – TA100); ^d^ mitomycin (0.5 μg/ plate – TA102), in the absence of S9 and ^e^ 2-anthramine (1.25 μg/ plate – TA 97a, TA98, TA100); ^f^ 2-aminofluorene (10.0 μg/ plate – TA102), in the presence of S9.

### 2.2. Antimutagenic Activity

The results obtained from studies on the antimutagenic potential of Bd-EAE and ArtC are presented in Table 2. The results are expressed as mean number of revertants/ plate (M), the standard deviation (SD) and the percent inhibition of mutagenic activity of a sample containing a mixture of mutagen and Bd-EAE or mutagen and ArtC, relative to the mutagenicity of the mutagen alone.

When strain TA98 was used in association with NOPD, a moderate antimutagenic effect was observed for the *B. dracunculifolia *extract, while ArtC showed a strong inhibitoty effect (42%). In experiments with metabolic activation for strain TA98, the antimutagenic activity of Bd-EAE against mutations induced by B[*a*]P was similar to that of ArtC: the mutagenicity of B[*a*]P was significantly reduced in a dose-dependent manner by 30 to 60% for Bd-EAE, and 38 to 62% for ArtC.

The Bd-EAE and ArtC did not reduce mutagenesis induced by SA, in the absence of metabolic activation, when strain TA100 was used. However, both Bd-EAE and ArtC did inhibit mutation induced by the alkylating agent AFB_1_ in TA100. The highest observed percent inhibition of mutagenicity (85%) was achieved with Bd-EAE, using strain TA100 in the presence of AFB_1_.

No reduction in the number of revertant colonies was observed for mutations induced by MMC in strain TA102. Moreover, Bd-EAE was not antimutagenic or produced insignificant decreases in the mutagenicity of AF in TA102, but ArtC induced a strong, dose-dependent effect, with % inhibition from 41 to 55%.

Only in the strain TA97a did Bd-EAE show a better effect than ArtC. In TA97a, Bd-EAE reduced the mutagenicity of NOPD by 45% and of AA by 36%. The ArtC showed no antimutagenic activity, against either NOPD or AA, in this strain.

## 3. Discussion

Medicinal plants have traditionally been used worldwide for the treatment of various human diseases [21]. They have proved to be abundant sources of biologically active compounds, many of which have been used as lead compounds to develop new pharmaceuticals [22]. However, a drug candidate that is active in a mutagenicity test or that produces mutagenic metabolites by activation in a microsomal enzyme system will generally be discarded in favor of a backup candidate [23].

The mutagenic activity of Bd-EAE, extracted from *B.dracunculifolia* leaves, and of the major compound present in the leaves (ArtC), and their influence on the activities of known mutagenic agents were assessed by Ames test in this study. Bd-EAE and ArtC alone had no mutagenic effect on the strains tested, either in the presence or absence of metabolic activation. The absence of such an effect by Bd-EAE against *S. typhimurium *bacterial strains in the Ames assay is a positive step towards determining its safe use in traditional medicine. Considering the popular use of this plant and the promising chemopreventive activity of ArtC, a lack of mutagenic effect in bacterial systems is highly relevant.

**Table 2:** Antimutagenic activity expressed as the mean and standard deviation of number of revertants and percent inhibition by Bd-EAE and ArtC of direct (-S9) and indirect (+S9) mutagens, tested on strains TA98, TA100, TA102 and TA 97a of *S. typhimurium.*

	**Treatments**	**Number of revertants (M ± SD)/ plate and % of inhibition**							
		**TA 98**				**TA 100**			
	**µg/plate**	**− S9**	**% inhibition**	**+ S9**	**% inhibition**	**− S9**	**% inhibition**	**+ S9**	**% inhibition**
**Bd-EAE**	**Ctrol +**	**NOPD**		**B[*a*]P**		**AS**		**AFB_1_**	
		638 ± 30		444 ± 14		1115 ± 36		2200 ± 140	
	**11.4**	482 ± 41	24 *	312 ± 9	30 **	1126 ± 57	-	1517 ± 78	31 **
	**22.8**	453 ± 19	29 **	247 ± 10	44 ***	1178 ± 61	-	1571 ± 86	29 **
	**45.6**	466 ± 35	27 **	218 ± 5	51 ***	1152 ± 77	-	1467 ± 85	33 **
	**91.4**	449 ± 10	30 **	196 ± 4	56 ***	1123 ± 17	-	741 ± 62	66 ***
	**182.8**	480 ± 27	25 *	175 ± 4	60 ***	1186 ± 53	-	327 ± 44	85 ***
**ArtC**	**Ctrol +**	842 ± 34		498 ± 33		2189 ± 73		2171 ± 33	
	**0.69**	526 ± 12	37 **	309 ± 17	38 **	1833 ± 48	16 *	1595 ± 37	26 **
	**1.37**	520 ± 18	38 **	282 ± 23	43 ***	1957 ± 36	11 *	1639 ± 33	24 *
	**2.75**	484 ± 31	42 ***	218 ± 24	56 ***	1813 ± 24	17 *	1575 ± 11	27 **
	**5.49**	532 ± 27	37 **	217 ± 27	56 ***	1727 ± 39	21 *	1600 ± 38	26 **
	**10.99**	499 ± 26	41 ***	189 ± 26	62 ***	1885 ± 25	14 *	1507 ± 43	30 **
		**TA 102**				**TA 97a**			
	**µg/plate**	**− S9**	**% inhibition**	**+ S9**	**% inhibition**	**− S9**	**% inhibition**	**+ S9**	**% inhibition**
**Bd-EAE**	**Ctrol +**	**MMC**		**AF**		**NOPD**		**AA**	
		935 ± 18		1336 ± 24		884 ± 34		2312 ± 81	
	**11.4**	1666 ± 87	-	1302 ± 8	2*	672± 11	24 *	1816 ± 76	21 *
	**22.8**	1309 ± 56	-	1344 ± 29	-	613 ± 68	31 **	1731 ± 64	25 **
	**45.6**	1317 ± 72	-	1363 ± 27	-	633 ± 55	28 **	1659 ± 82	28 **
	**91.4**	1011 ± 76	-	1357 ± 9	-	486 ± 35	45 ***	1603 ± 27	31 **
	**182.8**	891 ± 48	-	1209 ± 5	9 *	488 ± 65	45 ***	1481 ± 63	36 **
**ArtC**	**Ctrol +**	1072 ± 25		1099 ± 27		1106 ± 23		1861 ± 23	
	**0.69**	1096 ± 19	-	645 ± 23	41 ***	876 ± 8	21 *	1603 ± 26	14 *
	**1.37**	1022 ± 18	5 *	595 ± 25	46 ***	862 ± 14	22 *	1617 ± 16	13 *
	**2.75**	994 ± 25	7 *	566 ± 17	48 ***	885 ± 19	20 *	1669 ± 19	10 *
	**5.49**	943 ± 17	12 *	488 ± 14	56 ***	933 ± 7	16 *	1620 ± 21	13 *
	**10.99**	879 ± 11	18 *	529 ± 20	52 ***	891 ± 17	19 *	1602 ± 17	14 *

Bd-EAE = ethyl acetate extract of *B. dracunculifolia *leaves; ArtC = artepillin C; M ± SD = mean and standard deviation; Ctrol + = positive Control; NOPD = 4 -nitro-*o*-phenylenediamine (10.0 μg/ plate – TA98 and TA97a); SA = sodium azide (1.25 μg/ plate – TA100); MMC = mitomycin (0.5 μg/ plate – TA102), in the absence of S9 and B[*a*]P= benzo[*a*]pyrene (1.0 μg/ plate – TA 98); AFB_1_ = aflatoxin B1 (0.5 μg/ plate – TA 100); AA = 2-anthramine (1.25 μg/ plate – TA 97a); AF = 2-aminofluorene (10.0 μg/ plate – TA102), in the presence of S9; * no antimutagenic effect (< 25% inhibition); ** moderate effect (25% - 40% inhibition); *** strong antimutagenic effect (> 40% inhibition).

In a previous study, we also observed that Bd-EAE itself was not mutagenic in the rat micronucleus assay [7]. Andrade *et al.* [24] observed that the administration of high single doses of Bd-EAE did not induce any genotoxic response in blood cells of Swiss mice. Recently, Monteiro Neto *et al.* [25] evaluated the genotoxic potential of ArtC, and the results showed that ArtC itself was not genotoxic in the mouse micronucleus and comet assays.

Generally, cancer begins after a mutational episode in a single cell which multiplies and then progressively transforms to malignancy in multiple stages through sequential acquisition of additional mutations. In view of the fact that these initial events are the underlying causes of the whole progression of carcinogenesis, their inhibition would be an efficient preventive measure [26].

The antimutagenic properties of *B.* *dracunculifolia* have been studied, mainly because it is the main plant source of green propolis [7,24,27,28]. The results strongly suggest that *B. dracunculifolia *leaf bud extracts might display biological activities similar to those described for propolis. As it is hard to standardize propolis, because its activity and chemical composition may vary according to geographic location and plant source [7,29], the *B. dracunculifolia *extracts might be successfully incorporated into pharmaceutical products [7].

In view of the promising results obtained in experiments with *B. dracunculifolia*, and considering that the extract is a complex mixture of several unknown organic compounds [1], the evaluation of isolated compounds is even more relevant.

In this study, Bd-EAE and ArtC exhibited a protective effect against the mutagenicity induced by direct and indirect acting mutagens in the Ames test. They showed antimutagenic potential in more than one test strain and acted against various mutational mechanisms. NOPD, SA and MMC (direct mutagens), and B[*a*]P, AFB_1_, AA and AF (indirect mutagens) were included among these mutagens. 

In general, inhibitors of mutagenesis can act in one of several ways: By inhibiting the interaction between genes and biochemically reactive mutagens; inhibiting metabolic activation of indirectly acting mutagens by inactivation of metabolizing enzymes, or interacting with the pro-mutagens making them unavailable for the enzymatic process [30].

In the present study, Bd-EAE and ArtC demonstrated antimutagenic properties against frameshift mutations induced by the direct mutagen NOPD in the TA98 strain: Bd-EAE showed a moderate protective effect of 30% inhibition and ArtC showed a strong protective effect of 42% inhibition. In the TA97a strain, Bd-EAE reduced mutagenicity of NOPD by 45%, but ArtC showed no antimutagenic activity in this strain. The *S. typhimurium *test strain TA97a detects frameshift mutations in C-C-C-C-C-C; +1 cytosine and TA98 frameshift in DNA target-C-G-C-G-C-G-C-G [31].

The protection of the bacterial genome against directly acting mutagens may be due to the rapid elimination of mutagens from bacteria before their interaction with the DNA [32]. Bd-EAE and ArtC may facilitate or stimulate the bacterial transmembrane export system to eliminate the mutagens; they may also interfere with the uptake of mutagens into bacteria [32,33]. Bd-EAE and ArtC did not affect the SA-induced mutagenicity in strain TA100 and MMC in strain TA102.

Shimizu *et al.* [11] demonstrated that when ArtC reaches the liver, it stimulates the production of glutathione-S-transferase, an enzyme involved in cellular defenses against reactive oxygen compounds, as well as inducing the expression of phase II enzymes, which are responsible for the detoxification of carcinogens and, consequently, the suppression of precancerous lesions. This might explain the results obtained in the Ames test with metabolic activation, especially if we consider also that the biological activities of *B. dracunculifolia* are mostly due to its high levels of ArtC.

Bd-EAE and ArtC interacted with active intermediates, such as B[*a*]P, a well-known environmental procarcinogen, in tests on strain T98 with metabolic activation. The mutagenicity of B[*a*]P was significantly reduced in a dose-dependent manner by 30 to 60% by Bd-EAE, and 38 to 62% by ArtC.

B[*a*]P, a typical polycyclic aromatic hydrocarbon, is formed during incomplete combustion of organic matter and is a prevalent environmental pollutant. The levels of B[*a*]P in normal air range from 0.1 to 66 ng/m^3^, but occupational exposure during industrial and other domestic activities can increase these levels to 49 μg/m^3^. B[*a*]P has been detected in food, ranging from <0.1 to 7.2 μg/kg and in drinking water, ranging from 0.2 to 1,000 ng/L. The routes of human exposure to B[*a*]P are the ingestion of contaminated food and water and the inhalation of particulates in the ambient air and cigarette smoking [34].

Enzymatic activation of B[*a*]P by certain types of cytochrome P450 (CYP) found in the subcellular microsomal fraction, especially CYP1A1, is needed to produce the final carcinogen, (±)-benzo[*a*]pyrene-7,8-diol-9,10-epoxide [35]. This diol epoxide exerts its carcinogenic activity by alkylating nucleosides on DNA molecules at its bay region. The reaction occurs primarily with the purine bases, deoxyguanosine and deoxyadenosine, in DNA [36]. As a result, bulky stable and depurinating DNA adducts are formed [37,38]. Insufficient removal of these DNA adducts prior to replication creates hot spots in the gene and can result in deactivation of tumor suppressor genes or activation of oncogenes leading to tumor initiation [39,40].

There are at least two possible mechanisms through which Bd-EAE and ArtC can decrease B[*a*]P-DNA adduct formation, either by interacting with reactive intermediates or by interfering with the action of microsomal enzymes (e.g., CYP1A1) [40]. However, more studies are needed to confirm this hypothesis.

Bd-EAE and ArtC also reduced the frequency of mutations induced by the fungal toxin, AFB_1_, in TA100 with metabolic activation, resulting in the highest percent inhibition of mutagenicity (85%) attained with Bd-EAE. The *S. typhimurium *tester strain TA100, is capable of revealing base-pair-substitution point mutations.

Aflatoxins, a group of potent mycotoxins with mutagenic, carcinogenic, teratogenic, hepatotoxic and immunosuppressive properties, are of particular importance because of their adverse effects on animal and human health. Aflatoxins are produced as secondary metabolites by fungi of various species of *Aspergillus* (*A. flavus*, *A. parasiticus* and *A. nomius*) that grow on a variety of food and feed commodities. AFB_1_, which is the most toxic aflatoxin, is of particular interest because it is a frequent contaminant of many food products and one of the most potent naturally occurring mutagens and carcinogens known [41].

ArtC also induced a strong antimutagenic effect, significantly diminishing the mutagenicity of AF in TA102 with metabolic activation, in a dose-dependent manner, with 41 to 55% inhibition. No reduction in the number of revertant colonies induced by AF in the strain TA102 was observed with Bd-EAE. The *S. typhimurium *tester strain TA102 is normally used to detect cross-linking agents and base-pair-substitution mutations [31].

Alteration of the structure and function of P450 enzyme may result in altered rates and differential pathways of metabolism of mutagens and carcinogens, and provide protection against chemically-induced mutagenesis [30].

Other antimutagenicity studies have demonstrated that Bd-EAE and ArtC are able to protect against DNA damage induced by methyl methanesulfonate and doxorubicin, measured by micronucleus and comet assays [24,25].

Methyl methanesulfonate has been used for several decades as an experimental model to elucidate the mutagenic mechanisms of alkylating agents [25,42,43]. On the other hand, free radical production is considered the primary mechanism responsible for the toxicity of doxorubicin [44]. This results in oxidative stress and causes DNA damage, in turn leading to mutations and cancer development [25,45].

In this study, the antimutagenic property of Bd-EAE related to its ability to modulate the xenobiotic metabolizing enzymes in the liver, either by preventing the metabolic activation, or by altering the enzymatic activity in the detoxification pathway to induce the disposal of the known mutagen [26], was again illustrated by the results obtained with the mutagen 2-AA in strain TA97a with metabolic activation, where 35.9% inhibition was observed.

## 4. Experimental

### 4.1. Chemicals and Culture Media

Dimethylsulfoxide (DMSO), nicotinamide adenine dinucleotide phosphate sodium salt (NADP), D-glucose-6-phosphate disodium salt, magnesium chloride, L-histidine monohydrate, D-biotin, NOPD, SA, MMC, AA, AF, B[*a*]P and AFB_1_ were purchased from Sigma Chemical Co. (St. Louis, MO, USA). Oxoid Nutrient Broth Nº. 2 (Oxoid, England) and Difco Bacto Agar (Franklin Lakes, NJ, USA) were used as bacterial media. D-glucose, magnesium sulfate, citric acid monohydrate, anhydrous dibasic potassium phosphate, sodium ammonium phosphate, monobasic sodium phosphate, dibasic sodium phosphate and sodium chloride were purchased from Merck (Whitehouse Station, NJ, USA).

### 4.2. Preparation of *B. dracunculifolia* Extracts and Isolation of Artepillin C

Leaves of *B. dracunculifolia *De Candole were collected in Cajuru, São Paulo state, Brazil, in December 2005. The plant material was authenticated by Jimi N. Nakagima, and a voucher specimen (SPFR 06143) was deposited in the herbarium of the Biology Department of the University of São Paulo at Ribeirão Preto, São Paulo state, Brazil. Fresh plant material was air-dried at 40 °C for 48 h. The dried leaves (500.0 g) were powdered in a blender and submitted to maceration for 24 h in ethyl acetate at room temperature. The solvent was evaporated in a vacuum below 40 °C and 32.0 g of Bd-EAE was obtained [7]. ArtC was isolated from the hexane fraction of Brazilian green propolis, as described by Monteiro Neto *et al.* [25].

### 4.3. HPLC Analysis

Bd-EAE was submitted to HPLC analysis using the following equipment and conditions: Shimadzu high performance liquid-chromatograph (SCL-10A*vp *system controller, three LC-10AD pumps, SPD-M10A*vp *photodiode array detector and Shimadzu Class-VP 5.02 software). A CLC-ODS (M) column (4.6mm i.d. × 250 mm, 5-µm particle diameter) and a CLC G-ODS guard column were used as the stationary phase. The mobile phase had the following composition: A–B: 25–100% (B) in 60 min, A: 93.9% water, 0.8% acetic acid: 0.3% ammonium acetate: 5.0% methanol; B: acetonitrile; detection: 280 nm, flow rate of 1 mL/min. Most of the compounds detected were identified by comparison with authentic standards available at the Pharmacognosy laboratory, comparing UV spectra and considering both the maximum lambda and the relative area obtained with the use of two wavelengths (A_280/320_). The crude Bd-EAE was dissolved in methanol (HPLC grade) to obtain a concentration of 1 mg/mL. Before analysis, all samples were centrifuged at 1,300 rpm and filtered through a 45-µm filter [7].

### 4.4. Metabolic Activation System (S9 mixture)

The S9 fraction, prepared from livers of Sprague-Dawley rats treated with the polychlorinated biphenyl mixture Aroclor 1254 (500 mg/ kg), was purchased from Molecular Toxicology Inc. (Boone, NC, USA). The metabolic activation system consisted of 4% of S9 fraction, 1% of 0.4 M MgCl_2_, 1% of 1.65 M KCl, 0.5% of 1 M D-glucose-6-phosphate disodium and 4% of 0.1 M NADP, 50% of 0.2 M phosphate buffer and 39.5% sterile distilled water [46].

### 4.5. Mutagenicity Assay

Mutagenic activity was evaluated by the *Salmonella*/ microsome assay, using the *Salmonella typhimurium* tester strains TA98, TA100, TA97a and TA102, kindly provided by Dr. B.N. Ames (Berkeley, CA, USA), with (+S9) and without (−S9) metabolization by the pre-incubation method [46]. The strains from frozen cultures were grown overnight for 12–14 h in Oxoid Nutrient Broth No. 2. The metabolic activation mixture (S9) was freshly prepared before each test. For comparison of activity between Bd-EAE and ArtC, five different doses of the test compounds were assayed. All of them were diluted in DMSO. The concentrations of Bd-EAE were selected on the basis of a preliminary toxicity test. In all subsequent assays, the upper limit of the dose range tested was either the highest non toxic dose or the lowest toxic dose determined in this preliminary assay. Toxicity was apparent either as a reduction in the number of histidine revertants (His+), or as an alteration in the auxotrophic background (*i.e.*, background lawn). The concentrations varied from 11.4 to 182.8 µg/ plate for Bd-EAE and 0.69 to 10.99 µg/ plate for ArtC. The tested doses of ArtC were based on the corresponding concentration of this compound (about 6%) in Bd-EAE [25]. The various concentrations of Bd-EAE and ArtC to be tested were added to 0.5 mL of 0.2 M phosphate buffer, or to 0.5 mL of 4% S9 mixture, with 0.1 mL of bacterial culture and then incubated at 37 °C for 20–30 min. Next, 2 mL of top agar was added and the mixture was poured on to a plate containing minimal agar. The plates were incubated at 37 °C for 48 h and the His^+^ revertant colonies were counted manually. All experiments were analyzed in triplicate. The results were analyzed with the statistical software package Salanal (U.S. Environmental Protection Agency, Monitoring Systems Laboratory, Las Vegas, NV, version 1.0, from Research Triangle Institute, RTP, NC, USA), adopting the Bernstein *et al.* [47] model. The data (revertants/ plate) were assessed by analysis of variance (ANOVA), followed by linear regression. The mutagenic index (MI) was also calculated for each concentration tested, this being the average number of revertants per plate with the test compound divided by the average number of revertants per plate with the negative (solvent) control. A sample was considered mutagenic when a dose-response relationship was detected and a two-fold increase in the number of mutants (MI ≥ 2) was observed with at least one concentration [48]. The standard mutagens used as positive controls in experiments without S9 mix were NOPD (10 µg/ plate) for TA98 and TA97a, SA (1.25 µg/ plate) for TA100 and MMC (0.5 µg/ plate) for TA102. In experiments with S9 activation, AA (1.25 μg /plate) was used with TA98, TA97a and TA100 and AF (10 μg/ plate) with TA102. DMSO served as the negative (solvent) control (50 µL/ plate).

### 4.6. Antimutagenicity Assay

The antimutagenicity assay was conducted by means of the same procedure as the mutagenicity assay, except that Bd-EAE and ArtC were associated with known mutagens in tests with and without metabolic activation. In these tests, the direct-acting mutagens were 10.0 µg/ plate of NOPD (for *S. typhimurium* TA98 and TA97a), 1.25 µg/ plate of SA (for *S. typhimurium* TA100) and 0.5 µg/ plate of MMC (for *S. typhimurium* TA102), in the assay without metabolic activation, and the indirect-acting mutagens were 1.0 μg/ plate of B[*a*]P (for *S. typhimurium* TA98), 0.5 μg/ plate of AFB_1_ (for *S. typhimurium* TA100), 1.25 μg/ plate of AA (for *S. typhimurium* TA97a) and 10 µg/ plate of AF (for *S. typhimurium* TA102), in the assay with metabolic activation. All the plates were incubated at 37 °C for 48 h, and the number of revertant colonies per plate was counted manually. The entire assay was performed in triplicate. The antimutagenicity results were expressed as percent inhibition (the ability of the compounds to inhibit the action of the known mutagen). This was calculated as follows:

Inhibition (%) = 100 − [(T/M) × 100]

where T is the number of revertant colonies in the plate containing mutagen and compounds, and M is the number of revertant colonies in the plate containing only the mutagen [26].

No antimutagenic effect was recorded when inhibition was lower than 25%, a moderate effect for a value between 25% and 40%, and strong antimutagenicity for values greater than 40% [49,50].

Cell viability was also determined for each antimutagenesis experiment to assess the potential bactericidal effect of the mutagens. A substance was considered cytotoxic when the bacterial survival was less than 60% of that observed for the negative control [49,51].

## 5. Conclusions

In general, the results of the present study showed that Bd-EAE and ArtC alone had no mutagenic effect on the strains tested, either in the presence or absence of metabolic activation. With respect to the antimutagenic effect, ArtC showed a similar activity to that of Bd-EAE in some strains of *S. typhimurium, *demonstrating that the antimutagenic activity of Bd-EAE can be partially attributed to ArtC. The cases in which ArtC either potentiated or did not affect the activity of mutagenic agents, while Bd-EAE showed inhibitory activity, may be explained in part by a synergy between compounds present in the extract. In fact the present results support the idea that the protective effect of whole plant extracts is due to the combined and/or synergistic effects of a complex mixture of phytochemicals, the total activity of which may result in health benefits. 

Moreover, in view of the above results and hypotheses, we can observe that the inhibition of mutagenesis is often complex and involves multiple mechanisms. These results emphasize that antimutagenic mechanisms among natural plant extracts cannot be generalized, and it is worthwhile investigating each of them independently.
